# The Impact of Substrate Temperature on the Adhesion Strength of Electroplated Copper on an Al-Doped ZnO/Si System

**DOI:** 10.3390/ma17204953

**Published:** 2024-10-10

**Authors:** Jiun-Yi Tseng, Wen-Jauh Chen, Ping-Hang Chen

**Affiliations:** 1 Graduate School of Materials Science, National Yunlin University of Science and Technology, 123 University Road, Section 3, Yunlin, Douliu 64002, Taiwan; jytseng@yuntech.edu.tw (J.-Y.T.); m10747006@yuntech.edu.tw (P.-H.C.); 2 JBAO Technology Ltd., 24 Chunshenhu East Road, Xiangcheng District, Suzhou 215131, China

**Keywords:** Al-doped ZnO (AZO), textured silicon, adhesion strength, electroplated copper

## Abstract

This research, which involved a comprehensive methodology, including depositing electroplated copper on a copper seed layer and Al-doped ZnO (AZO) thin films on textured silicon substrates using DC magnetron sputtering with varying substrate heating, has yielded significant findings. The study thoroughly investigated the effects of substrate temperature (Ts) on copper adhesion strength and morphology using the peel force test and electron microscopy. The peel force test was conducted at angles of 90°, 135°, and 180°. The average adhesion strength was about 0.2 N/mm for the samples without substrate heating. For the samples with substrate heating at 100 °C, the average peeling force of the electroplated copper film was about 1 N/mm. The average peeling force increased to 1.5 N/mm as the substrate heating temperature increased to 200 °C. The surface roughness increases as the annealing temperature of the Cu/AZO/Si sample increases. These findings not only provide a reliable and robust method for applying AZO transparent conductive films onto silicon solar cells but also underscore its potential to significantly enhance the efficiency and durability of solar cells significantly, thereby instilling confidence in the field of solar cell technology.

## 1. Introduction

Photovoltaics (PVs) have become a significant source of electricity in recent decades. Silicon heterojunction (SHJ) solar cells are gaining attention due to their high 26.81% conversion efficiency, excellent passivation, and process temperatures typically below 200 °C [1,2,3,4]. A typical silicon solar cell consists of multiple layers in a specific structure to efficiently convert sunlight into electrical energy. The structure includes a substrate, front contact (transparent conductive oxide, TCO), antireflection coating, emitter layer, base layer, back surface field (BSF), back contact, and passivation layer. Each of these components plays a crucial role in the overall performance and efficiency of the solar cell. Transparent conductive oxide (TCO) enables efficient light absorption, charge carrier collection, and electricity generation. Commonly used TCO materials include indium tin oxide (ITO), fluorine-doped tin oxide (FTO), and aluminum- or other element-doped zinc oxide (ZnO). These materials offer high transparency in the visible spectrum and good electrical conductivity, making them suitable for solar cells and other optoelectronic devices [5,6,7].

Indium tin oxide (ITO) has been commonly used as a transparent conductive oxide (TCO) in silicon solar cells. However, it has some drawbacks. Indium, a key component of ITO, is relatively rare and expensive. Indium-based materials may release toxic fumes or particles when processed or damaged, requiring careful handling and waste management procedures. To address these issues, it is essential to develop alternative TCO materials with improved properties such as abundance, cost-effectiveness, flexibility, durability, and environmental sustainability. Ongoing research aims to explore novel TCO materials and deposition techniques that can overcome ITO limitations and enhance the performance and reliability of silicon solar cells [5,6,7].

Here, we consider the potential of using zinc oxide doped with aluminum (ZnO: Al) to replace indium tin oxide (ITO) in various optoelectronic applications. Indium is rare and expensive, leading to price fluctuations and supply chain issues. On the other hand, zinc and aluminum are more abundant and cost-effective, making ZnO:Al potentially more economical for large-scale production. Additionally, the production and disposal of ITO involve environmental concerns due to indium’s limited availability and extraction methods. In contrast, ZnO:Al offers a more environmentally friendly option, as zinc and aluminum are more readily available and less environmentally impactful. ZnO:Al films also exhibit a good thermal stability, making them suitable for the high-temperature processing steps involved in device fabrication. This stability ensures that ZnO:Al-based devices maintain their performance under various operating conditions [8,9].

Electroplating copper technology is an exciting and proven alternative for creating electrodes for solar cells. Copper metallization is a promising technique for silicon heterojunction solar cells. Adachi et al. achieved a 25.1% efficiency with copper-plated SHJ solar cells [10]. In the structure of SHJ solar cells, a TCO film is used as a conductive layer and anti-reflection coating. Due to its low sheet resistance and high transparency, indium tin oxide (ITO) is a commonly used TCO in SHJ cells. This process involves depositing a layer of copper onto the surface of the silicon substrate to enhance the solar cell’s electrical conductivity and efficiency. Despite challenges such as cost and conductivity, copper-plated metallization for silicon solar cells offers potential benefits, such as improved performance and reliability, making it a technology worth exploring.

Copper-plated metallization for silicon solar cells shows potential but also presents several challenges. One challenge is the diffusion of copper into silicon, which is especially problematic at high temperatures during solar cell processing or operation. This diffusion can harm the silicon’s electrical properties, increasing recombination losses and reducing cell performance. Additionally, when copper comes into contact with silicon, it can form undesirable copper silicide compounds. These compounds can create high-resistivity regions within the solar cell structure, increasing series resistance and decreasing efficiency. Ensuring the good adhesion of copper metallization to the silicon substrate is also challenging, as poor adhesion can cause delamination or cracking of the metallization layer, compromising electrical contact and cell performance. Addressing these challenges requires advanced material engineering, process optimization, and a careful selection of materials and manufacturing techniques. Ongoing research efforts are focused on developing copper-plated metallization solutions that offer improved performance, reliability, and compatibility with silicon solar cell technology [11].

The adhesion between plated copper and the TCO layer presents a challenge for the process of the copper metallization of SHJ solar cells. Aguilar et al. compared the direct electroplating of Cu on different metal seeds (Ag, Ni, Cr, and Ti deposited on transparent conductive oxide by physical vapor deposition) on transparent conductive oxide to improve the adhesion of the ITO and the copper layer [12]. They found that directly electroplating Cu onto the sputtered Ag seed achieved the best adhesion, with the silver seed layer exhibiting more than 5 N/mm of pull force in a pull test. Our previous study found that the maximum and average peel force values for electroplating copper on Cu/AZO/Si at room temperature are 0.39 and 0.2 N/mm, respectively. These results were obtained through rigorous testing, including a pull test to measure adhesion strength. The results indicated weak adhesion between electroplating copper and AZO/Si [13]. It is essential to address the weak adhesion between electroplating copper and AZO/Si if AZO is introduced to the SHJ solar cell, underscoring the necessity for further research and development.

The properties of thin films are influenced by the substrate temperature, as demonstrated by various experimental studies. Sivaji Reddy investigated how substrate temperature affects the structural, electrical, optical, and morphological properties of ITO thin films and their impact on the current–voltage characteristics of OLED [14]. Zhang et al. discovered that substrate temperature plays a crucial role in the microstructure, optical, and electrical phase transition properties of VO_2_ films [15]. Kim et al. illustrated the significance of substrate temperature in determining the structural, compositional, and optical properties of Cu_2_O films synthesized via RF-magnetron sputtering [16]. Ghos et al. reported on the effects of substrate temperature, sonication process, and post-annealing on the structural, morphological, and optical properties of ZnO thin films grown using the successive ionic layer adsorption and reaction (SILAR) method [17]. Lastly, this paper examines the impact of substrate temperature on the mechanical properties of electroplating copper on Cu/AZO/Si.

## 2. Materials and Methods

A single crystal phosphorus-doped silicon wafer with a pyramid texture was used as the substrate. The textured silicon substrate was cleaned with acetone and H_2_SO_4_/H_2_O_2_ solutions. The substrate was dipped into a hydrogen fluoride solution before being loaded into the sputtering vacuum system to obtain the fresh layer of the silicon. The AZO and copper seed films were sputter-deposited onto textured silicon substrates in a DC/RF-magnetron sputtering system. The base pressure before sputtering was 2 × 10^−7^ Torr. An AZO target was used to prepare the AZO films by using an RF power held at 60 W during deposition in an ambient argon environment of 99.999% purity. A fixed argon flow rate was maintained at 50 SCCM, and the operation pressure was 6 × 10^−3^ Torr. During the copper film deposition, the constant argon flow rate was 25 SCCM, and the DC power was held at 30 W.

**Scheme 1:**
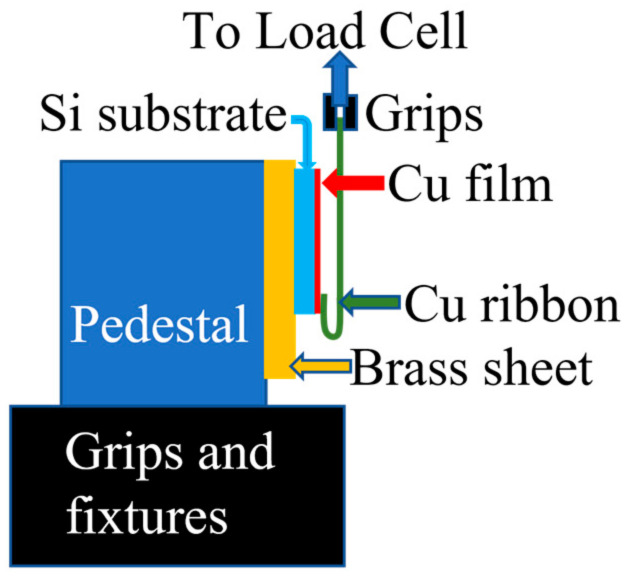
Schematic drawing of the peel test.

The textured silicon substrate temperatures were 25, 100, and 200 °C during the sputtering of the AZO and copper films. The sputtered AZO and copper films were 65 and 50 nm thick, respectively. After the AZO films had been deposited, copper films were sputter-deposited in the same vacuum chamber. For the above samples, those without substrate heating (25 °C) and substrate heating at 100 and 200 °C are designated as Cu/AZO/Si(RT), Cu/AZO/Si(100), and Cu/AZO/Si(200), respectively. Cu/AZO/Si(X) represents the Cu/AZO/Si(RT), Cu/AZO/Si(100), and Cu/AZO/Si(200) samples. Then, further electroplated copper was added to three samples. A chemical bath was used for the electrodeposition of copper onto three samples. The reagent-grade chemicals and deionized water were used to prepare the chemical bath solution, and a 300 mL beaker was used as the electroplating cell. Magnetic stirrer agitation was applied during electroplating, and the bath temperature was set to 25 °C. The time taken for the electroplating of the copper layer is about 25 min. The Cu/AZO/Si(X) electroplated with copper metals is designated as EP-Cu/Cu/AZO/Si(X). A JEOL scanning electron microscope (SEM, JSM-7610F Plus, JEOL, Tokyo, Japan) equipped with EDS (Energy Dispersive X-ray Spectrometer) operated at 20 kV to examine surface morphologies and identify elements. A focus ion beam (FIB, FEI Quanta 3D FEG, FEI, Hillsboro, OR, USA) was used to prepare a cross-sectional view of the SEM. An electron beam within the FIB chamber formed a thin Pt layer to protect the sample surface. A focused beam of gallium ions was used to mill the sample ion to give a cross-section view. The scanning electron microscope (SEM) operated at 20 kV. A universal testing machine was used for the peel force test. The schematic drawing of the peel test is shown in Scheme 1. Three specimens per sample were tested. The test samples were glued to a brass sheet. The Sn layer coated on Cu ribbons (40 mm × 1.5 mm × 0.15 mm) was hand-soldered on a sample with a 4 mm width. The peel-off tests were conducted at three angles (90°, 135°, and 180°). The speed was 30 mm/min. The measured values were divided by 4 mm to obtain the real peel force.

## 3. Results

**Figure 1:**
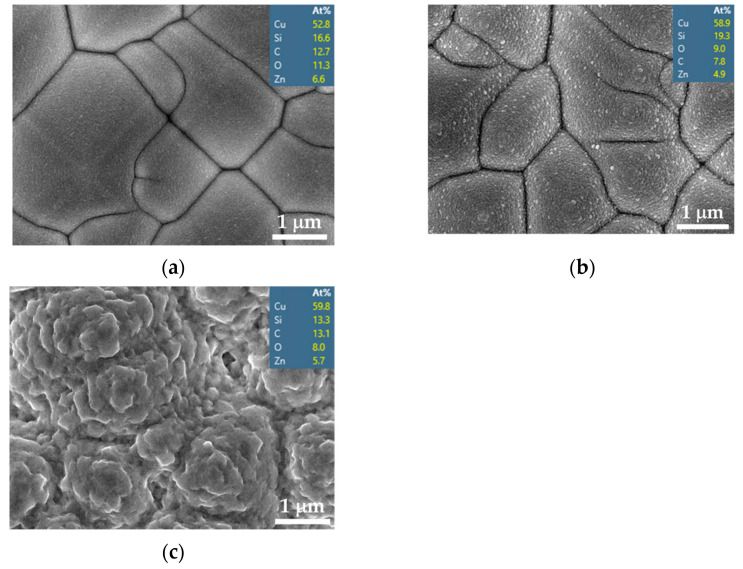
The SEM images of (**a**) the as-deposited sample, (**b**) Cu/AZO/Si(100), and (**c**) Cu/AZO/Si(200).

The surface morphologies of the Cu/AZO/Si samples, both as-deposited (RT) and annealed at temperatures of 100 and 200 °C, are depicted in Figure 1. The surface appears smooth for the as-deposited sample (Figure 1a). Minor fluctuations were observed on the surface of the samples annealed at 100 °C. At 200 °C, the surface resembles a cauliflower, displaying an increased roughness with higher annealing temperatures. The surface roughness of the Cu/AZO/Si(100) sample is slightly higher than that of the Cu/AZO/Si(RT) sample. However, the surface of the Cu/AZO/Si(200) sample is substantially rougher compared to the Cu/AZO/Si(RT) and Cu/AZO/Si(100) samples.

One of the EDS results for the semi-quantitative analysis of elements is included in Figure 1a–c for the Cu/AZO/Si(RT), Cu/AZO/Si(100), and Cu/AZO/Si(200) samples, respectively. The oxygen-to-copper ratio can be calculated after removing the oxygen content in the ZnO. The ratios of oxygen to copper are 8.7%, 6.0%, and 3.2% for the Cu/AZO/Si(RT), Cu/AZO/Si(100), and Cu/AZO/Si(200) samples, respectively. These ratios decrease as the annealing temperature increases, indicating a reduction in surface copper oxide with higher substrate heating temperatures.

**Figure 2:**
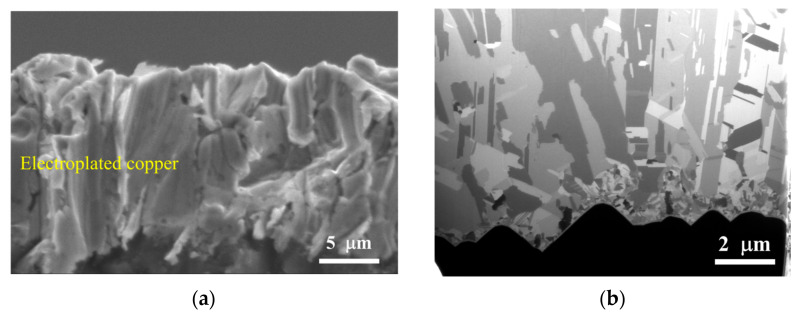
SEM cross-sectional image of the EP-Cu/Cu/AZO/Si(RT) sample prepared by (**a**) cleavage of the test sample and (**b**) FIB.

Figure 2 shows the SEM cross-sectional views of the EP-Cu/Cu/AZO/Si(RT) sample. The image in Figure 2a is of the cleavage of the test sample. The thickness of the Cu film is approximately 15 µm (Figure 2a). The image shown in Figure 2b was obtained by FIB. The grain of electroplated copper has a long shape with a width of about 1 μm (Figure 2b).

**Figure 3:**
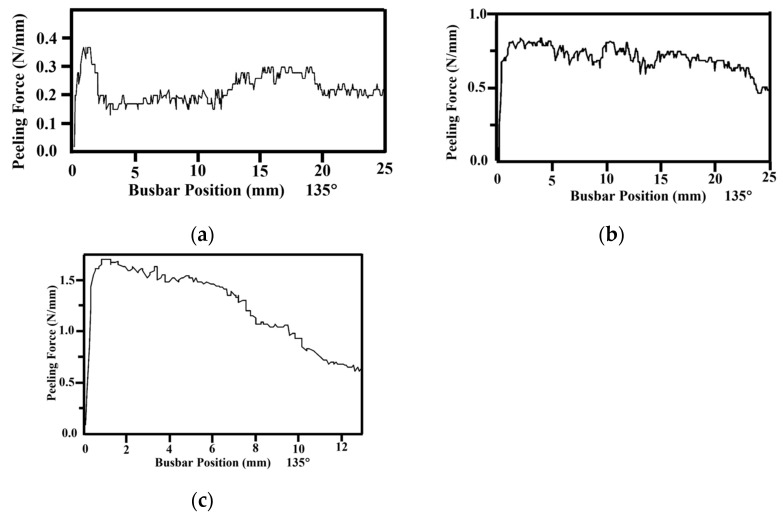
Peel force diagrams for the (**a**) EP-Cu/Cu/AZO/Si(RT), (**b**) EP-Cu/Cu/AZO/Si(100), and (**c**) EP-Cu/Cu/AZO/Si(200) samples, which were tested at an angle of 135° with a constant speed of 30 mm/min.

In Figure 3a–c, the peel force diagrams for the EP-Cu/Cu/AZO/Si(RT), EP-Cu/Cu/AZO/Si(100), and EP-Cu/Cu/AZO/Si(200) samples are illustrated. These samples were tested at an angle of 135° with a constant speed of 30 mm/min. The maximum peel force values of 0.38, 0.8, and 1.7 N/mm were recorded for the samples in Figure 3a room temperature and substrate heating at Figure 3b 100 °C and Figure 3c 200 °C, respectively. Additionally, average peel force strengths of 0.2, 0.75, and 1.25 N/mm were obtained for the EP-Cu/Cu/AZO/Si(RT), EP-Cu/Cu/AZO/Si(100), and EP-Cu/Cu/AZO/Si(200) samples, respectively.

It Is worth noting that the maximum peel force values increased from 0.38 N/mm for EP-Cu/Cu/AZO/Si(RT) to 1.7 N/mm for EP-Cu/Cu/AZO/Si(200). The maximum peel force value of EP-Cu/Cu/AZO/Si(200) is approximately four times higher than that of the EP-Cu/Cu/AZO/Si(RT) samples. The average peel force drops from 1.25 N/mm for EP-Cu/Cu/AZO/Si(200) to 0.2 N/mm for the sample without substrate heating. These findings indicate that higher substrate heating temperatures increase the maximum and average peel force.

**Figure 4:**
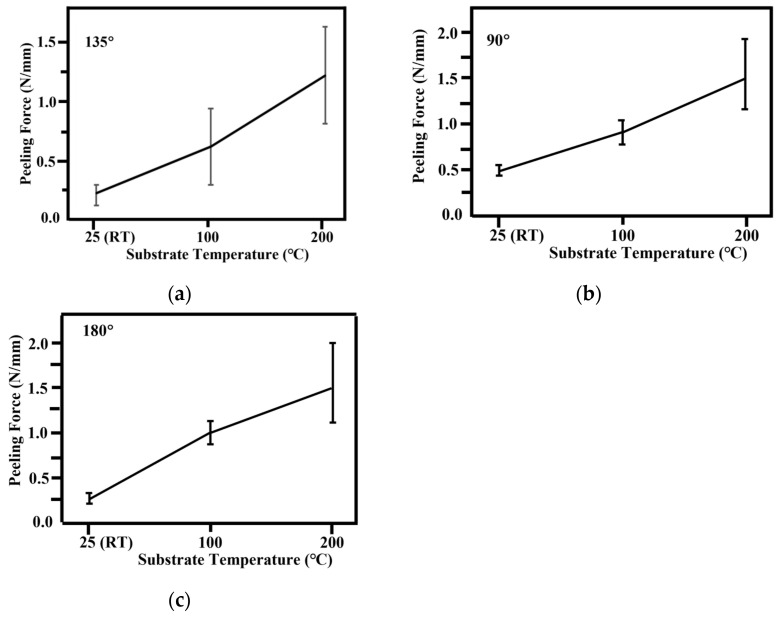
The relationship between peel force and substrate heating at test angles of (**a**) 135°, (**b**) 90°, and (**c**) 180°.

Figure 4a–c illustrates the effect of substrate heating on the peel force test at angles of 135°, 90°, and 180°. When the peel force test was carried out at angles of 90°, the average peel force values were 0.5, 0.8, and 1.5 N/mm, and for angles of 180°, the values were 0.23, 1.0, and 1.5 N/mm. The peel forces increase as the substrate temperature rises at test angles of 90°, 135°, and 180°, indicating that adhesion strength improves with higher substrate temperatures. Substrate heating can enhance adhesion during AZO and copper seed layer deposition. The impact of substrate heating is evident in the peel force exceeding 1 N/mm at a temperature of 200 °C.

**Figure 5:**
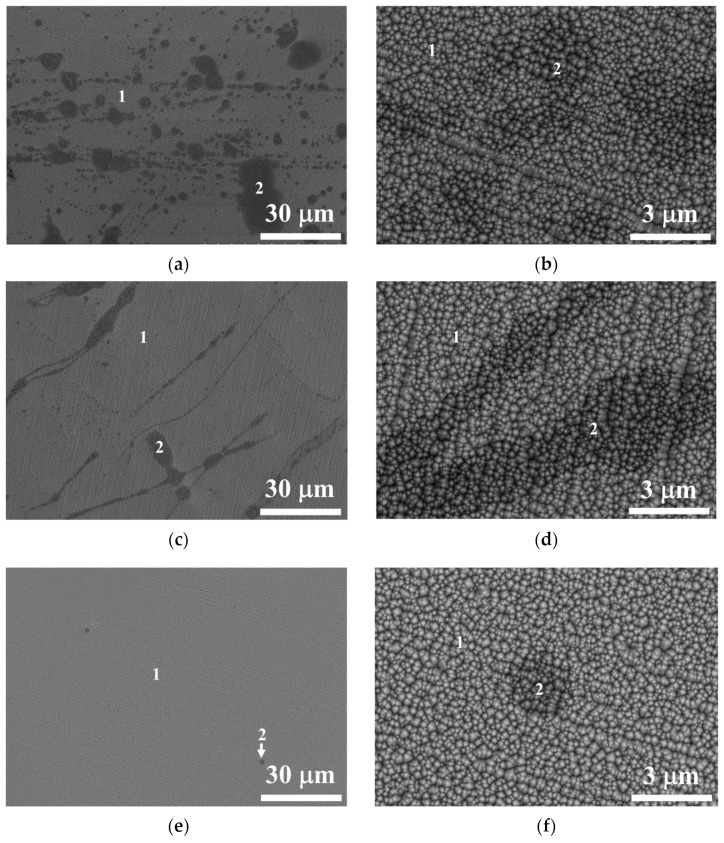
The surface morphologies of the substrates after the peel test for the as-deposited EP-Cu/Cu/AZO/Si sample (**a**,**b**) and the samples annealed at 100 °C (**c**,**d**) and 200 °C (**e**,**f**). The arrow indicates the location of area 2 in Figure 5e.

The surface morphologies were also examined after the peel test. Figure 5a,c,e show low-magnification SEM images for the EP-Cu/Cu/AZO/Si(RT), EP-Cu/Cu/AZO/Si(100), and EP-Cu/Cu/AZO/Si(200) samples after the peel test, respectively. The high magnification SEM images shown in Figure 5b,d,f are for the EP-Cu/Cu/AZO/Si(RT), EP-Cu/Cu/AZO/Si(100), and EP-Cu/Cu/AZO/Si(200) samples. A gray zone (zone 1) and a black zone (zone 2) can be observed for all samples. The area of the black zone is smaller than that of the gray zone for all samples. Furthermore, the area of the black zone decreases as the substrate temperature increases. The EP-Cu/Cu/AZO/Si(200) sample only shows a small black zone.

**Figure 6:**
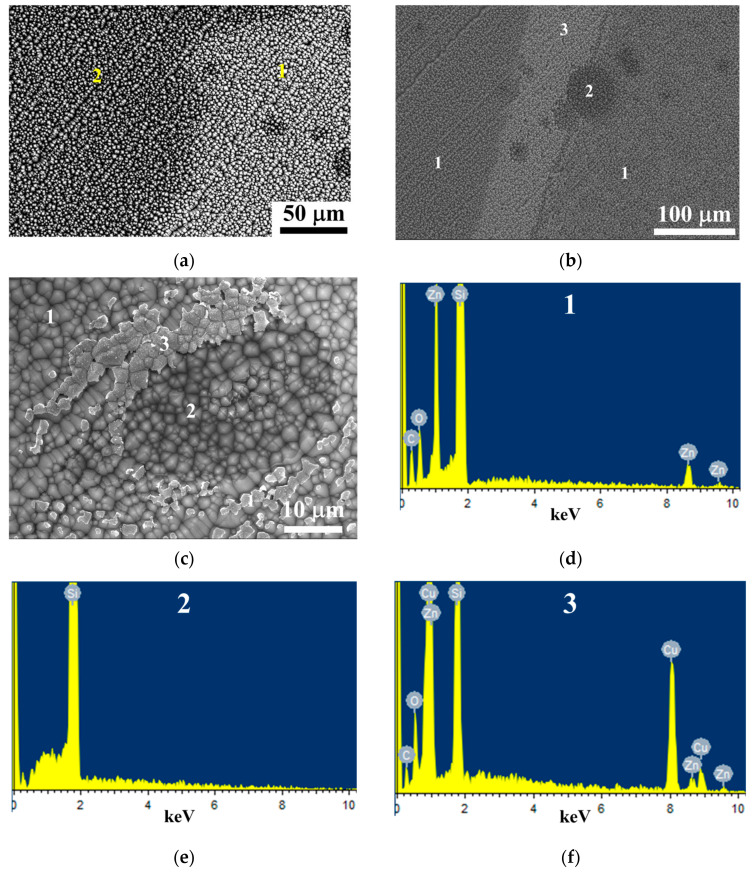
SEM images for the (**a**) EP-Cu/Cu/AZO/Si(RT), (**b**) EP-Cu/Cu/AZO/Si(100), and (**c**) EP-Cu/Cu/AZO/Si(200) samples after peel test. The numbers 1, 2, and 3 in (**a**–**c**) represented the zones 1, 2, and 3 of morphology. EDS spectrum for (**d**) zone 1, (**e**) zone 2, and (**f**) zone 3.

The chemical composition of all samples was determined using the energy-dispersive X-ray spectrum (EDS, Oxford Link). Figure 6a–c show the SEM images for the EP-Cu/Cu/AZO/Si(RT), EP-Cu/Cu/AZO/Si(100), and EP-Cu/Cu/AZO/Si(200) samples after the peel test, respectively. There are two zones in the image of the EP-Cu/Cu/AZO/Si(RT) sample (Figure 6a). Numbers 1 and 2 represent the area of zone 1 and zone 2, respectively. The EP-Cu/Cu/AZO/Si(100) and EP-Cu/Cu/AZO/Si(200) samples have zone 1, zone 2, and zone 3. The EDS spectrum of zones 1, 2, and 3 is shown in Figure 6d–f, respectively. Zone 1 has Si, Zn, and O elements in the gray area. Zone 2 (black) contains only Si. Zone 3 can be observed in the EP-Cu/Cu/AZO/Si(100) and EP-Cu/Cu/AZO/Si(200) samples, where Si, Zn, Cu, and O elements emerge.

## 4. Discussion

The peel test is a simple and quick method to assess the adhesion of interconnector ribbons to solar cell metallizations. This method is part of the solar cell DIN standard [18] and is widely accepted due to its ease of evaluating cell metallizations and the soldering process [19]. A force of 1 N per millimeter of joint width is required. According to Figure 4, the peel forces are 1.5, 1.25, and 1.5 N/mm for the EP-Cu/Cu/AZO/Si(200) sample test at angles of 90°, 135°, and 180°, respectively. The EP-Cu/Cu/AZO/Si(200) sample meets the standard DIN EN 50461 criteria, exhibiting high contact adhesion above 1 N/mm. However, the peel forces are consistently below 1 N/mm for the EP-Cu/Cu/AZO/Si(RT) and EP-Cu/Cu/AZO/Si(100) samples tested at 90°, 135°, and 180°. This suggests that heating the substrate can increase the adhesion strength of the EP-Cu/Cu/AZO/Si system, making it feasible to introduce AZO into the silicon solar cell. The surface roughness increases as the annealing temperature of the Cu/AZO/Si sample increases, as shown in Figure 1. The peeling force also increases as the substrate temperature increases, and the increased surface roughness due to substrate heating plays an important role.

Kluska et al. conducted a significant study on Al-BSF solar cells, using a unique process to analyze the impact of silicidation on electrical and mechanical contact adhesion [20]. The peel testing was meticulously performed at a 90° angle to the contact. They carried out laser patterning by UV laser ablation with nanosecond (LCO 1) and picosecond (LCO 2) durations and laser chemical processing (LCP). The results of the peel test of the unannealed specimen reveal significant insights. The median and maximum peel forces of LCO 1 are less than 0.1 N/mm and 0.2 N/mm, respectively, indicating a lack of substantial contact adhesion in this process. The median and maximum peel forces in LCP are slightly higher than those for LCO 1 at 0.2 N/mm and 0.3 N/mm, respectively. Samples with LCO 2 exhibited the highest average peel strength, with median and maximum peel forces of 0.6 and 1.3 N/mm, respectively. The research by Kluska et al. underscores the importance of plating contacts, demonstrating the most significant peeling force due to the increased surface roughness of the picosecond laser treatment. Rehman et al. also contributed to the field by reporting that Ni surface etching could be adopted to enhance the Ni–Cu interface adhesion [21]. Laser-ablated and photolithography-based wet chemical etching was used to pattern samples for the pull tab adhesion test. The author performed the peel test at an angle of 180° and a constant speed of 30 mm/min. The results showed that the average peel forces of the samples prepared by laser ablation and photolithography were 1.5 ± 0.2 N/mm and 0.33 ± 0.06 N/mm, respectively. This study is significant as it demonstrates that the lithography technique, which is wet chemically etched and produces a smoother surface, results in low peeling forces. Conversely, with their rough surface, laser-ablated samples exhibit higher adhesion strengths. Laser-ablated samples with rougher surfaces have higher adhesion strengths than samples patterned with photolithography-based wet chemical etching. The research by Kluska et al. and Rehman et al. also highlights the high peeling force due to the increased surface roughness.

Ulrich Eitner and Li Carlos Rendler used Kinloch’s mechanical theory to convert measured peel forces into adhesive fracture energies [19]. The median peel forces at angles of 90°, 135°, and 180° were 3.07 N, 2.35 N, and 3.39 N, respectively. Based on the test results, the peel force at a 135° angle was lower than at 90° and 180°. According to Figure 4, the peel forces for the EP-Cu/Cu/AZO/Si(200) sample were 1.5 N/mm, 1.25 N/mm, and 1.5 N/mm at angles of 90°, 135°, and 180°, respectively. For the EP-Cu/Cu/AZO/Si(100) sample, the peel forces were 0.8 N/mm, 0.75 N/mm, and 1 N/mm at angles of 90°, 135°, and 180°, respectively. Our findings also revealed that the peel force at a 135° angle was lower than at 90° and 180°.

In Figure 6, we see that zone 1 (gray area) contains elements of Si, Zn, and O, while zone 2 (black) only contains Si. The analysis indicates delamination between copper and AZO in zone 1 and silicon and AZO in zone 2. Additionally, zone 3 shows that the fracture occurs in the copper layer.

Only zones 1 and 2 are visible in the EP-Cu/Cu/AZO/Si(RT) sample. The peel forces are below 0.5 N/mm, suggesting a weak interface between AZO, silicon, and copper.

The adhesion strength of the interface between AZO and silicon and copper and AZO increases as the substrate temperature rises to 100 °C. The peel force reaches 1 N/mm when tested at a 180° angle. As the substrate temperature increases to 100 °C, the black area (zone 2) decreases, indicating that the increase in adhesion strength of the interface between AZO and silicon is higher than the increase in the interface between copper and AZO.

Raising the substrate temperature to 200 °C enhances the adhesion strength of the interface between AZO and silicon and copper and AZO. At this temperature, the peel force reaches 1.5 N/mm when tested at a 180° angle. The black area (zone 2) nearly disappears at 200 °C, indicating that the increase in the adhesion strength of the interface between AZO and silicon is more significant than the increase in the interface between copper and AZO.

For EP-Cu/Cu/AZO/Si (200), most of the delamination occurs at the interface between copper and AZO. The adhesion strength interface between AZO and silicon significantly increases for EP-Cu/Cu/AZO/Si(200). Zone 3 can be observed in both EP-Cu/Cu/AZO/Si(100) and EP-Cu/Cu/AZO/Si(200), suggesting that some copper rupture can be observed in these cases.

The results show that heating the substrate can improve the adhesion strength of the interface between AZO and silicon and copper and AZO. The peel forces of the EP-Cu/Cu/AZO/Si(200) sample meet the solar cell standard DIN EN 50461’s criteria.

## 5. Conclusions

The results show that increasing the substrate temperature enhances the adhesion strength of the interface between AZO and silicon and between copper and AZO. At a 180° peel angle, samples’ average peel force values at room temperature, with substrate heating at 100 °C and 200 °C, were 0.23, 1.0, and 1.5 N/mm, respectively. The peel forces increase as the substrate temperature rises at all test angles. The peel force at a 135° angle is also lower than that at 90° and 180°.

The EP-Cu/Cu/AZO/Si(RT) sample has a weak interface between AZO and silicon and copper and AZO. The adhesion strength of these interfaces increases as the substrate temperature rises. Notably, the increase in adhesion strength at the interface between AZO and silicon surpasses that between copper and AZO, marking a significant advancement in our understanding. Moreover, the adhesion strength of the interface between AZO and silicon and copper and AZO increases as the substrate temperature reaches 200 °C. The EP-Cu/Cu/AZO/Si(200) sample shows the most delamination at the interface between copper and AZO. Importantly, copper can be observed in the EP-Cu/Cu/AZO/Si(100) and EP-Cu/Cu/AZO/Si(200) samples, suggesting some copper rupture.

The results demonstrate that substrate heating can increase the adhesion strength of the interface between AZO and silicon and between copper and AZO. Furthermore, the peel forces of the EP-Cu/Cu/AZO/Si(200) sample meet the solar cell standard DIN EN 50461’s criteria.

## Data Availability

The original contributions presented in the study are included in the article. Further inquiries can be directed to the corresponding authors.
